# Summary of two questionnaires designed to understand the research climate for Bioimage Analysts in the UK between 2016-2019

**DOI:** 10.12688/f1000research.51794.1

**Published:** 2021-04-06

**Authors:** Dominic Waithe

**Affiliations:** 1 WIMM Centre for Computational Biology, University of Oxford, Oxford, OX3 9DS, UK

**Keywords:** bioimage analysis, community, questionnaire, career

## Abstract

**Background:** Bioimage analysis is an emerging field within the global research community. It is an interdisciplinary discipline which requires knowledge of biology, image analysis and biophysics. This report represents the analysis and discussion of two questionnaires run by the Image Analysis Focused Interest Group of the Royal Microscopical Society (IAFIG-RMS). The goal of this document, which represents the analysis and interpretation of these questionnaires, is to highlight the current research climate for Bioimage Analysts in the UK and discusses some of the problems and possibilities for this emerging discipline.

**Methods:** Two questionnaires (2016 and 2019) were developed and sent to researchers in the UK using mailing lists and forums specific for microscopy and image analysis. The participants were asked a range of questions spanning different aspects of their work and funding. Respondents were collected and analysed using Jupyter notebooks.

**Results: **The analysis of the responses from these questionnaires highlighted many interesting issues and aspects of this community. It is clear that a major issue for the community is the nature of the funding and the long-term career possibilities available. Furthermore, the issue of independence is discussed with clear evidence that researchers would like to pursue their own research with the option of dedicated time to support the research of others.

**Conclusions:** It is our hope that this study will help catalyse funding opportunities which help support this emerging discipline and help it establish a unique identity for itself within the research community in the UK and beyond.

## Introduction

A Bioimage Analyst is a research scientist who performs, as their primary role, image analysis in the context of biomedical research. This broadly involves developing pipelines of analysis which can be used to extract data from images acquired from microscopy. This role is discrete from general image analysis or computer vision due to the close integration it has with biological and biophysical research practises. Schemes like Neubias have done an excellent job of highlighting the need for Bioimage Analysts and have also provided good training opportunities for them ^1, 2^ across the EU and beyond. Unfortunately, however, the Neubias EU Cost funded scheme has now ended and although multiple aspects of the scheme will continue, it is up to individual countries, universities and funders to develop this movement further. This support includes providing career opportunities in universities which support this role (e.g. lectureships, professorships, facility roles) and also for the research bodies to fund these roles at multiple levels (e.g. fellowships, core funding, project grants).

In the UK research bodies and universities in the UK have not yet addressed the unique nature of this group of people and often researchers feel their careers have been side-lined as a result. To address this, two questionnaires were designed and distributed. The initial questionnaire was centred on the theme "Do you use or develop bioimage analysis for life sciences research?". In the second questionnaire we then focused on the issue of funding within the community of bioimage analysis and drill down further into this subject. In summary, we show that there is an active community of scientists who are performing image analysis in terms of their own research and for the benefit of others. The following document discusses the points raised.

## Methods

The questionnaires were distributed through mailing lists specific to microscopy and image analysis, and addressed to the UK readership of these lists. Questionnaire 1 was distributed to the following mailing lists (on the specified dates): ImageJ mailing list ( IMAGEJ@LIST.NIH.GOV)(23/09/2016), UK-Eurobioimaging-project ( uk-eurobioimaging-project@jiscmail.ac.uk) (23/09/2016), Confocal Microscopy mailing list ( LISTSERV@LISTS.UMN.EDU)(23/09/2016), Royal Software Engineers ( everyone@rse.ac.uk) (23/09/2016). An additional email was sent to the Image World mailing list ( sci-diku-imageworld@list.ku.dk) (29/09/2016). No follow-up emails were sent. 89 of the 99 respondents had submitted official UK academic email addresses. No submissions were excluded. In questionnaire 1 a Wordcloud (1) was created from the job title entries using the website: https://www.wordclouds.co.uk/. using an online resource For Questionnaire 2 a UK specific microscopy image analysis mailing list was available (IAFIG Jiscmail) and used to distribute the emails: Two emails were sent to this list ( IAFIG-RMS@jiscmail.ac.uk) (10/01/2019 and 16/01/2019). In addition, the questionnaire was promoted through a tweet on Twitter (community response: 22 Retweets, 6 Tweets, 13 Likes), and also with a post on the Image.sc image analysis web forum ( https://forum.image.sc). Furthermore an email was forwarded to the UK-Eurobioimaging-project ( uk-eurobioimaging-project@jiscmail.ac.uk)(11/01/2019). 65 of the 84 respondents submitted official UK academic email addresses. No submissions were excluded. 24 of the original 99 participants of Questionnaire 1 returned and answered Questionnaire 2.

Both questionnaires were designed and run using the website SurveyMonkey ( https://www.surveymonkey.co.uk).

Please refer to the *Extended data* ^3^ for details of the questions asked for each Questionnaire. Responses were analysed using the scripting language Python (3.7.4) and were analysed and visualised using Jupyter lab notebooks.

### Ethical considerations

Due to the non-sensitive nature of the content, in addition to the lack of identifying information being included in this publication, no ethical approval was sought for this study. None of the data presented here, or in the accompanying Zenodo repository, can be used to identify survey participants, and the data is not accessible through the SurveyMonkey platform used to host the questionnaires ( https://www.surveymonkey.com/mp/policy/privacy-policy/). Completion of the questionnaire after reading the information sheet was taken as consent to participate in the survey.

## Results and discussion

Both questionnaires have proved highly informative.

### Questionnaire 1

Many researchers who identify as being Bioimage Analysts have developed the necessary expertise for themselves having responded to a need in the community. As such they often come from a diverse range of roles and are classified according to their employers in a variety of different ways. When we asked scientists who perform bioimage analysis, "What is your current job/position title?" we got a very broad distribution of answers.

**Figure 1.**
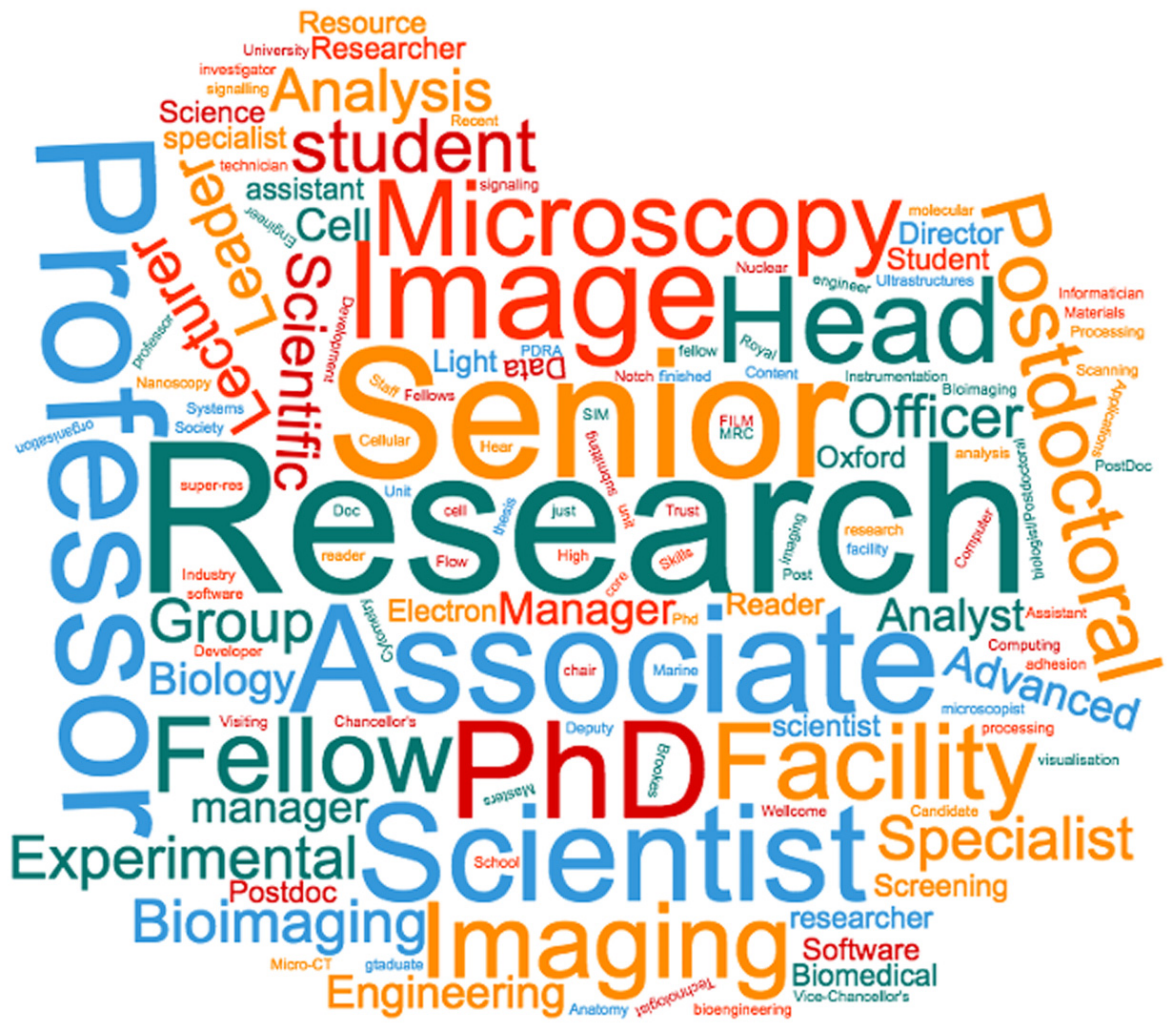
A "Word Cloud" representing the answers from Q1.1. Participants were able to submit one or more words describing their job position/title. Each word was treated independently.

***Q1.1: What is your current job/position title?*** Out of the 99 people that answered the first questionnaire only a maximum of three people gave the same answer to the above question and very few people gave the answer ‘Image Analyst‘ and no-one gave the answer ‘Bioimage Analyst‘. The above ‘Word Cloud Image‘ ( Figure 1) gives an idea of the range of nomenclature for individuals in this discipline. Going by their job title it suggests that bioimage analysis is not the main focus of their work.

**Figure 2.**
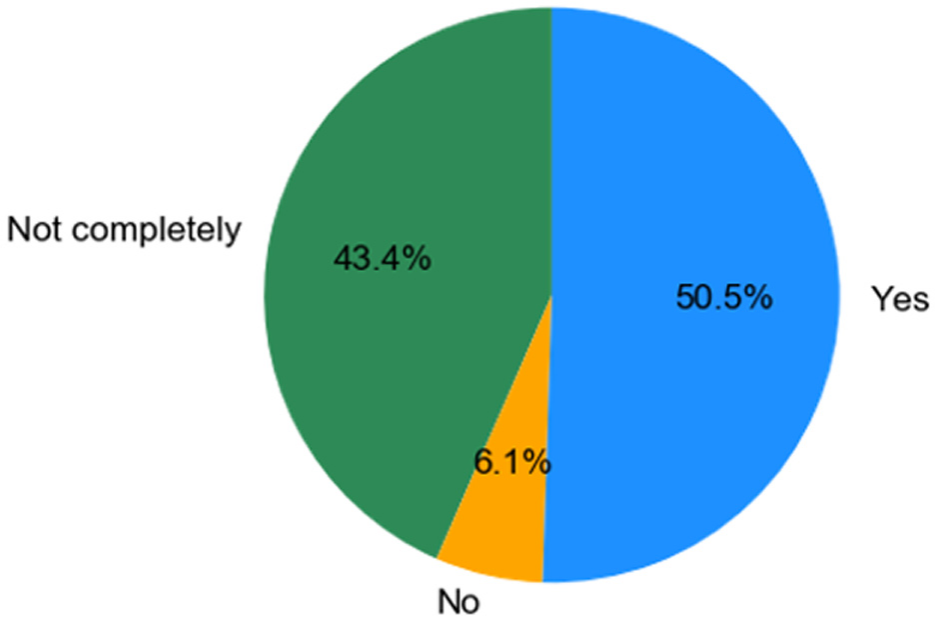
Pie chart representing answers from Q1.2. Participants were able to select only one answer.

***Q1.2: Do you feel that your current job/position title accurately describes the work that you do?*** In Figure 2 we show the outcome from asking: "Do you feel that your current job/position title accurately describes the work that you do?" To this question 50.5% of individuals said ’YES’ to the above question whereas around 43.4% said ’Not completely’ and 6.1% said ’No’, not at all. This suggests that there is a large component of individuals for whom their job title and description doesn’t quite describe the focus of their work. This doesn’t prove that they should be named ’Image Analyst’ or similar but it does suggest individuals are assuming or taking on roles which are not reflected by their job-title. As we see later, there is evidence to suggest these individuals are mostly performing image analysis.

**Figure 3.**
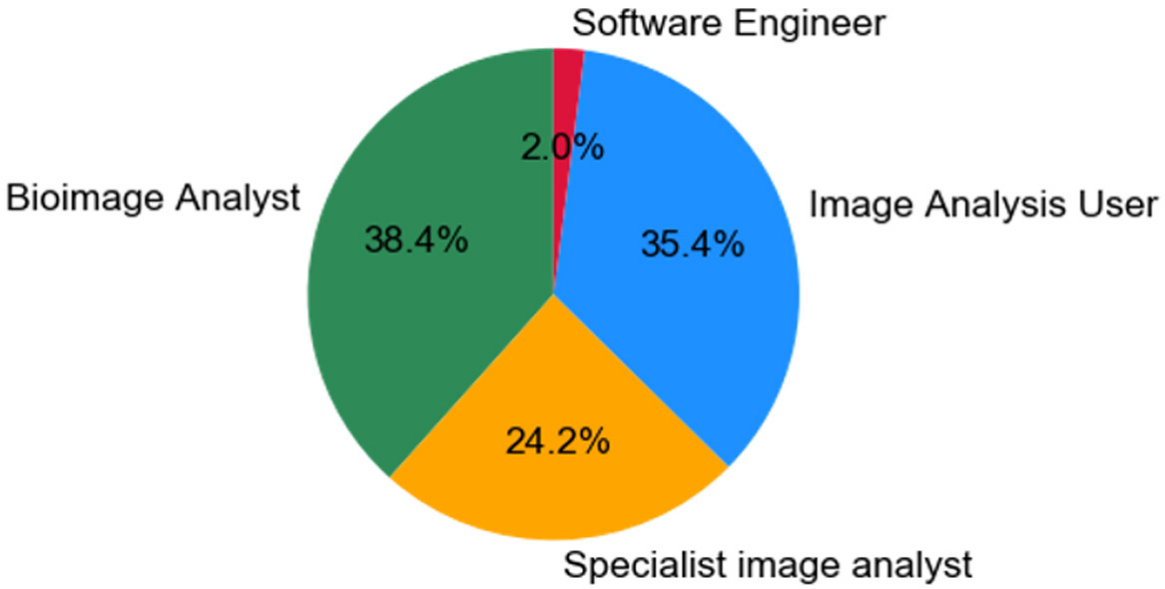
Pie chart representing answers from Q1.3. Participants were able to select only one answer.

***Q1.3: Which of the following categories best describes your working relationship with image analysis?*** We posed the question, "Which of the following categories best describes your working relationship with image analysis?". The responses to this question were very interesting ( Figure 3). In total, 35.4% saw themselves as ’Image Analysis Users’. These are individuals who treat image analysis as a tool that they use in their research. Although potentially skilled, they do not create or develop analysis methods, algorithms or pipelines. Many of these researchers are likely laboratory bioscientists interested in using image analysis tools for testing biological hypotheses, without an active role in developing algorithms and pipelines. The remaining 64.6% of individuals put themselves in a more specialist role, mostly either as a Bioimage Analyst, Specialist Image Analyst but a few used the term Software Engineers. This essentially means that 64.6% of the individuals who filled out this questionnaire have a professional level of image analysis expertise and are employed in the life sciences. Along with the first two questions, it suggests that these individuals in their roles are not defined according to the actual job they are performing, but have found themselves in these roles as means to do what they want, or what is needed (i.e. image analysis). In our follow-up questionnaire (section 2, Figure 7 and Figure 8) we asked the same question but provided more focused answers and analysis.

**Figure 4.**
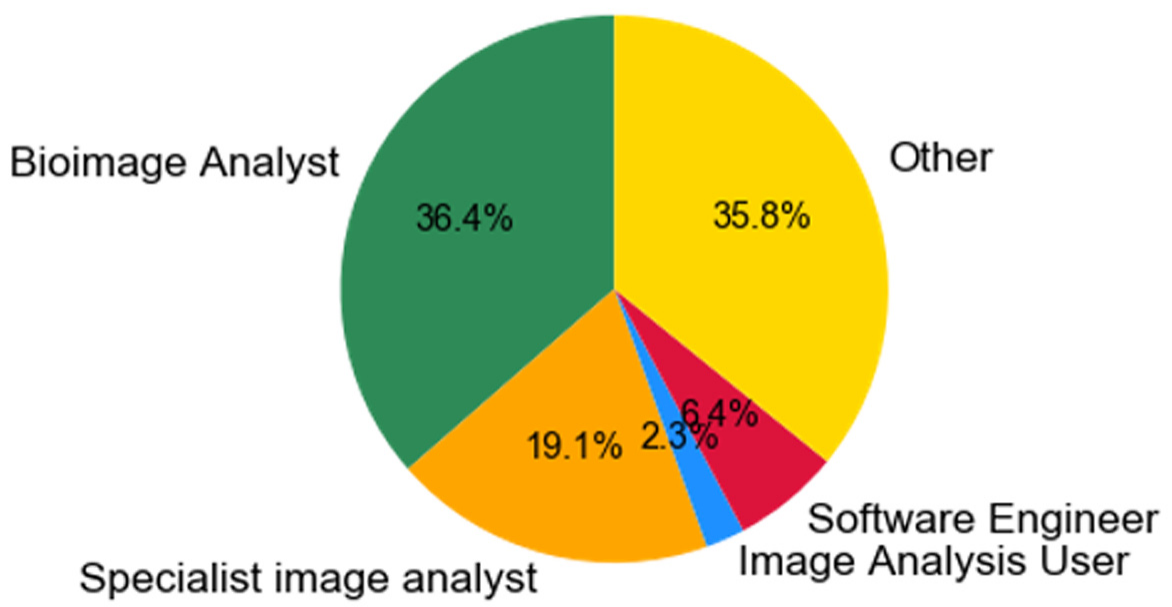
Pie chart representing answers from Q1.4. Participants were able to select multiple answers.

***Q1.4: Often we perform different roles at different times. how much of your time, on average, do you commit to the following activities?*** For this question subjects were asked how they organise their day-to-day research between the activities of a Bioimage Analyst, Software Engineer, Image Analysis User and other research activities. We weighted people’s answers depending on whether they stated that they spent all of their time (weight = 8), most of their time (6), half of their time (4), just some (1) or none of their time (0) performing the specified activities. Broadly speaking most individuals spent most of their time performing some kind of image analysis, whilst compacting all other activities into a smaller proportion of their time ( Figure 4). This is very interesting as it shows that most of the individuals who filled out this questionnaire are indeed Image Analysts and that other forms of research and experimentation are secondary to their data analysis.

**Figure 5.**
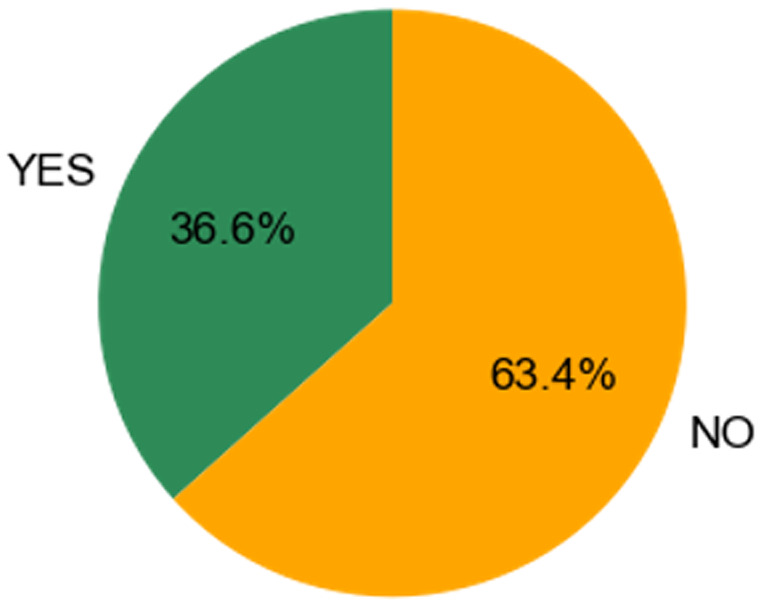
Pie chart representing answers from Q1.5. Participants were able to select only one answer.

***Q1.5: If you are interested in pursuing image analysis as potential career, do you feel as though there are sufficient options for career progression in your chosen field of academia?*** Figure 5 shows the distribution to answers from the following question: "If you are interested in pursuing image analysis as potential career, do you feel as though there are sufficient options for career progression in your chosen field of academia?" The distribution of answers to this question is very clear. Of those individuals interested in pursuing image analysis 63.4% of them said that they did not have sufficient career options to develop in this direction. This essentially means that although these individuals are ful-filling an important and high-demand area of bioscience research, there is not a sufficient framework in place to support their specific career development. The previous questions prove that many individuals are performing image analysis as the dominant aspect of their day-to-day research, even if it isn’t their obvious job description.

We reasoned that the issues affecting the bioimage community highlighted in the first questionnaire relate ultimately to the organization of funding in the UK. In our follow-up questionnaire (questionnaire 2), we asked a number of questions relating to the funding of scientists that perform some kind of bioimage analysis in their work. Through this characterization (84 respondents) we have been able to clarify the funding sources which are funding bioimage analysis and are now in a position to make suggestions to the community as a whole.

### Questionnaire 2

**Figure 6.**
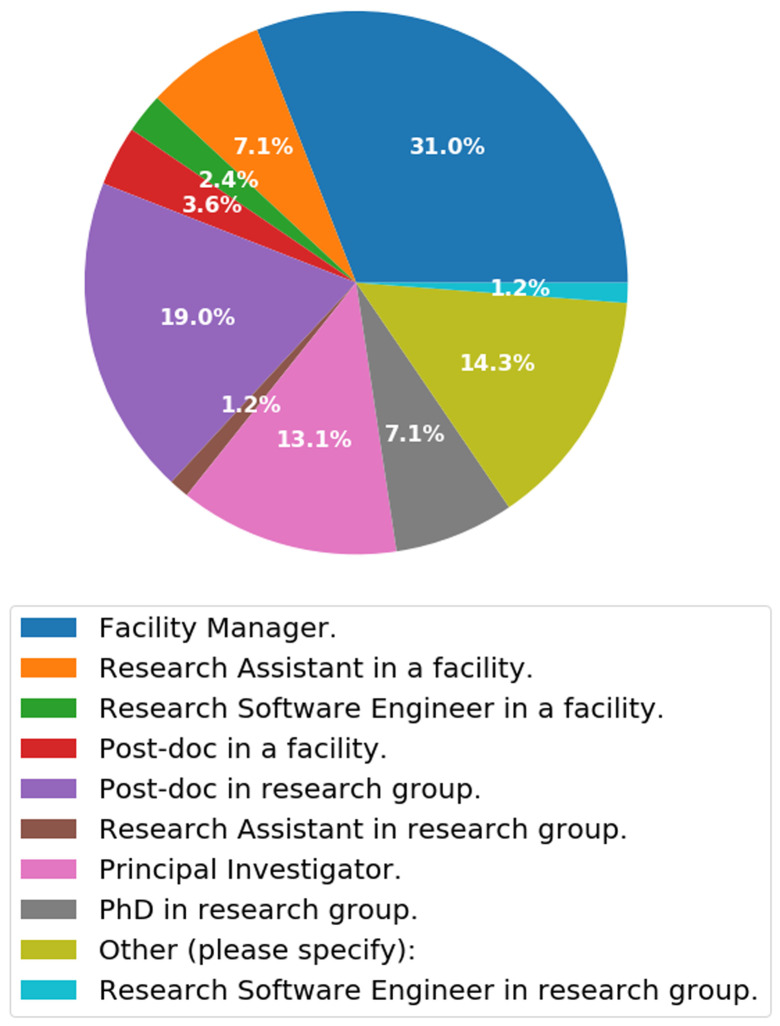
Pie chart summarising answers from Q2.3. What best describes your position? Participants were able to select only one answer.

***Q2.3: What best describes your position? (Please choose the closest role).*** Q2.3 ( Figure 6) was designed to find the closest job description which scientists will classify themselves into. Previously we showed the diversity of titles that people will assign to themselves in (Q1.1), but here we ask specifically which title best describes their position. At the moment, the category that is most dominant among those doing bioimage analysis is Facility Manager (31%), followed by post-docs in a research group (19%). This is to be as expected as scientists, from within a facility, will need to assist researchers by providing image analysis expertise, whereas post-docs will be performing analysis to pursue their own research or developing new techniques for the community. If we compare the ratio of facility-based staff (44.1%) to research group staff (41.5%) who are doing some form of bioimage analysis, the ratio is about 50:50. Therefore a simplified view of this is that from those that filled out questionnaire approximately 50% of scientists are on an academic career track and the other 50% can be considered research technical professionals.

**Figure 7.**
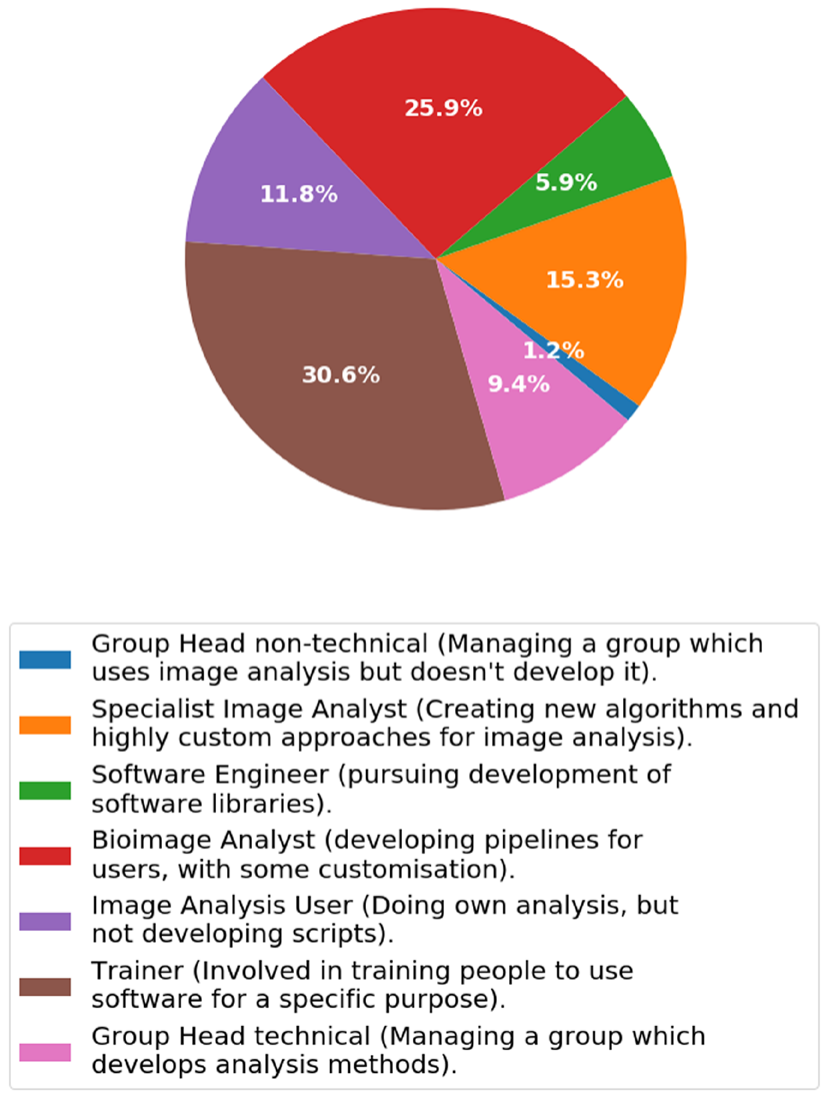
Answers from Q2.4 pooled amongst facility associated staff (e.g. Facility Manager, Research assistant in a facility, Research Software Engineer in a facility, Post-doc in a facility). Participants were able to check multiple options.

**Figure 8.**
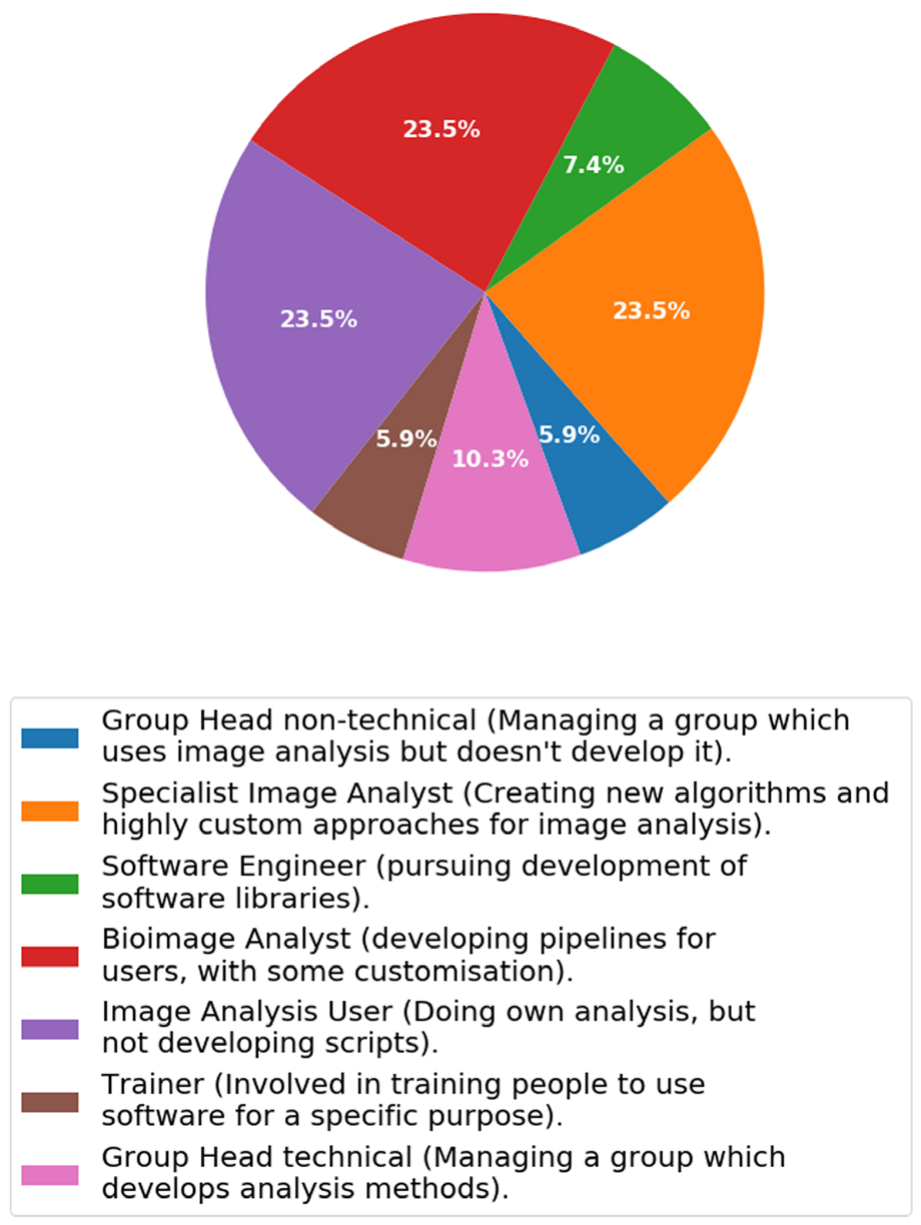
Answers from Q2.4 pooled amongst research group associated staff (e.g. Principal Investigator, Research Assistant in a research group, Research Software Engineer in a research group, PhD, Post-doc in a Research Group). Participants were able to check multiple options.

***Q2.4: Which of the following categories best describes your working relationship with image analysis?*** We asked the participants to describe their relation with image analysis ( Figure 7, Figure 8) by checking the categories they most associated with the role. We took the answers and pooled those individuals whom associated mostly with facilities and compared it to those who predominantly associated with research groups as determined from Q2.3. Figure 7 and Figure 8 show that there are some clear differences between the two cohorts.

The main differences between the work relating to bioimage analysis between a facility and a research group is that much more training is being performed in the facility, whereas the ’specialist image analyst’ and also the ’Image Analysis Users’ are more abundant in research groups. The proportion of individuals performing training in research groups (5.6%) is low compared to those performing training in a facility (30.6%). Conversely however there are more ‘Image Analysis Users’ in research groups (facilities: 11.8%, research groups: 23.5%) and also more ‘Specialist image analysts’ (facilities: 15.3%, research groups: 23.5%). This shows that research groups are more interested in applying algorithms through software (as an Image Analysis User) and/or developing specialist algorithms and approaches. Interestingly however the amount of bioimage analysis is consistent amongst the groups (facilities: 25.9%, research groups: 23.5%) showing that connecting pipelines of algorithms is a common goal, whether it be to support users or to develop novel pipelines of analysis.

**Figure 9.**
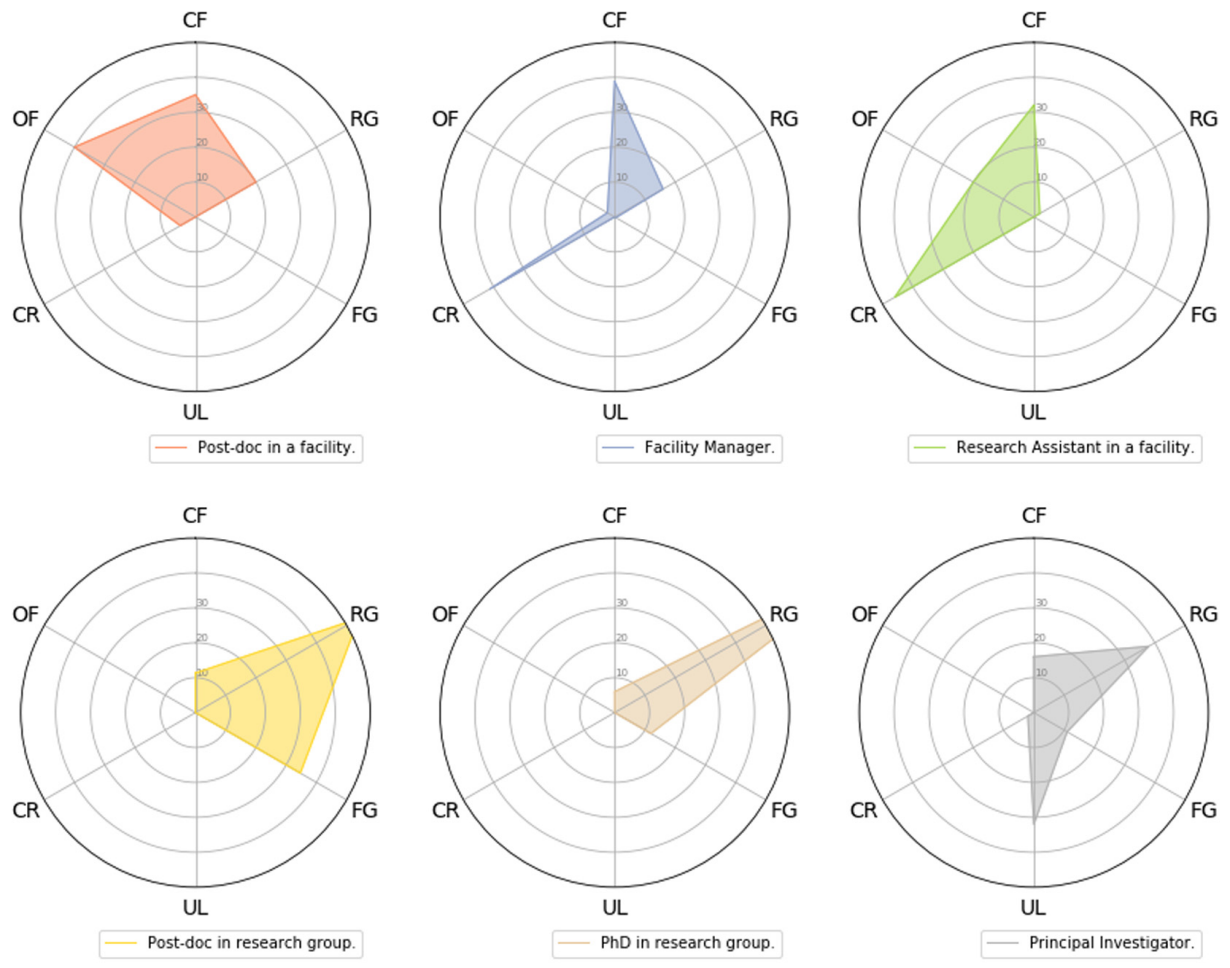
Radial plots displaying answers to question 2.5. Participants were asked to gauge the approximate funding contributions (0–100%) of their position from the following sources: CF (Core Facility), RG (Research Grants), UL (University Lectureship), FG (Fellowship Grant), CR (External and Internal Cost Recovery), OF (Other funding). Center, 0% funding, Outside Circle, 100%, each graduation represents 20%. Values in each category were averaged over each class of participant.

***Q2.5: Where does your funding come from? (0–100)%*** The academic positions (i.e. PhD positions, post-doc, PI positions) attract mainly funding from research grants, fellowships, with some PIs being partially funded by lectureships ( Figure 9). Facility managers and research assistant positions are funded predominantly by cost-recovery and core-funding. This is fairly typical in the life sciences where facility and technical staff are mainly funded by core-funding or funding generated within an institute whereas researchers are attracting funding mainly from research grants

**Figure 10.**
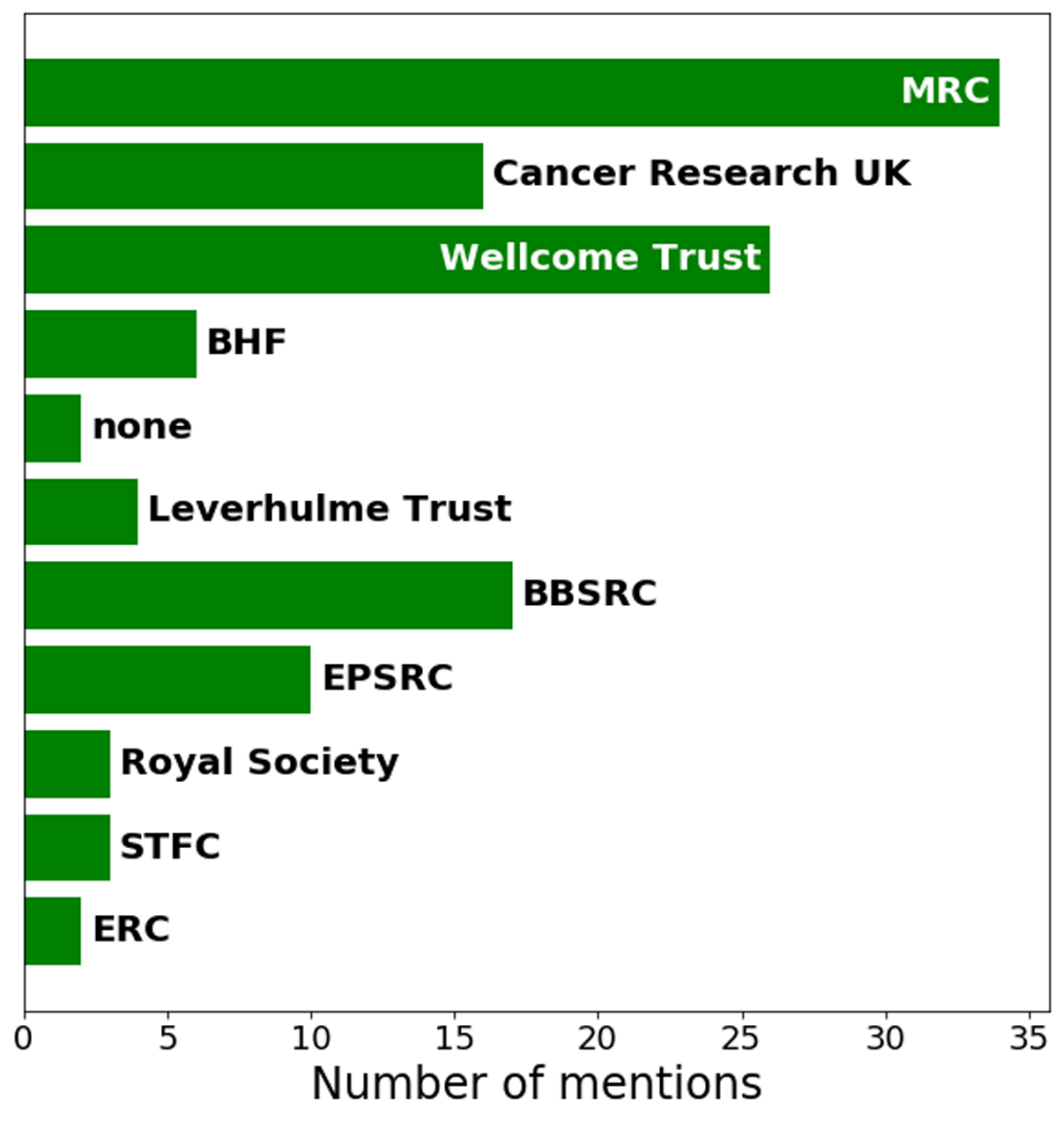
Bar chart visualising answers to Q2.6. Participants were able to check multiple options.

***Q2.6: Which UK Research Councils or Charities fund your research?*** From the answers generated from Q2.6 it is clear the main funders for bioimage analysis at the moment are the MRC, BBSRC ^1^, EPSRC, Wellcome and CRUK ( Figure 10). This result is fairly consistent with the idea that the research grants are indirectly funding bioimage analysis research through conventional funding routes.

**Figure 11.**
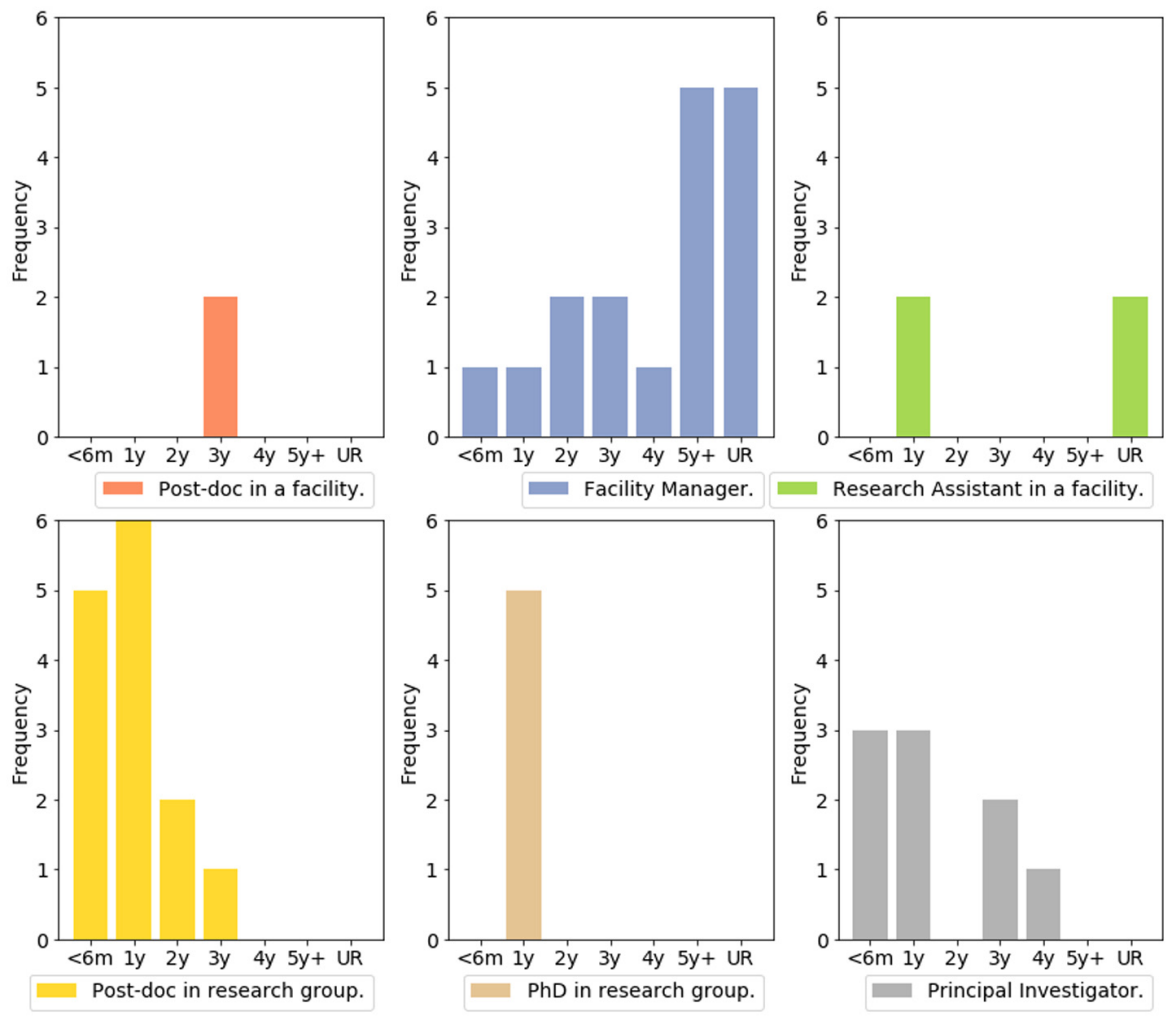
Bar charts representing funding horizon for different job types, answers from Q2.6. Categories represent: <6m (< 6 months), 1y (1 year), 2y (2 years), 3y (3 years), 4y (4 years), 5+ (5 years and more), UR (Until retirement).

***Q2.7: How much longer will your current funding last? (If you have multiple funding sources, choose the one that runs out first).*** The more academic posts (post-docs, PhDs, PI) tend to have a more narrow funding horizon, whereas the facility roles and research assistant roles can (not always) benefit from longer funding ( Figure 11). This is shown by the apparent skew of distributions toward the left-hand side in the categories of Post-doc in research group, PhD in research group and PI. This highlights the known career dynamic that influences research and facility positions. Facility positions often mean your academic career is limited, but can often mean you have access to better job security, a factor which influences many researchers whom take up these posts.

**Figure 12.**
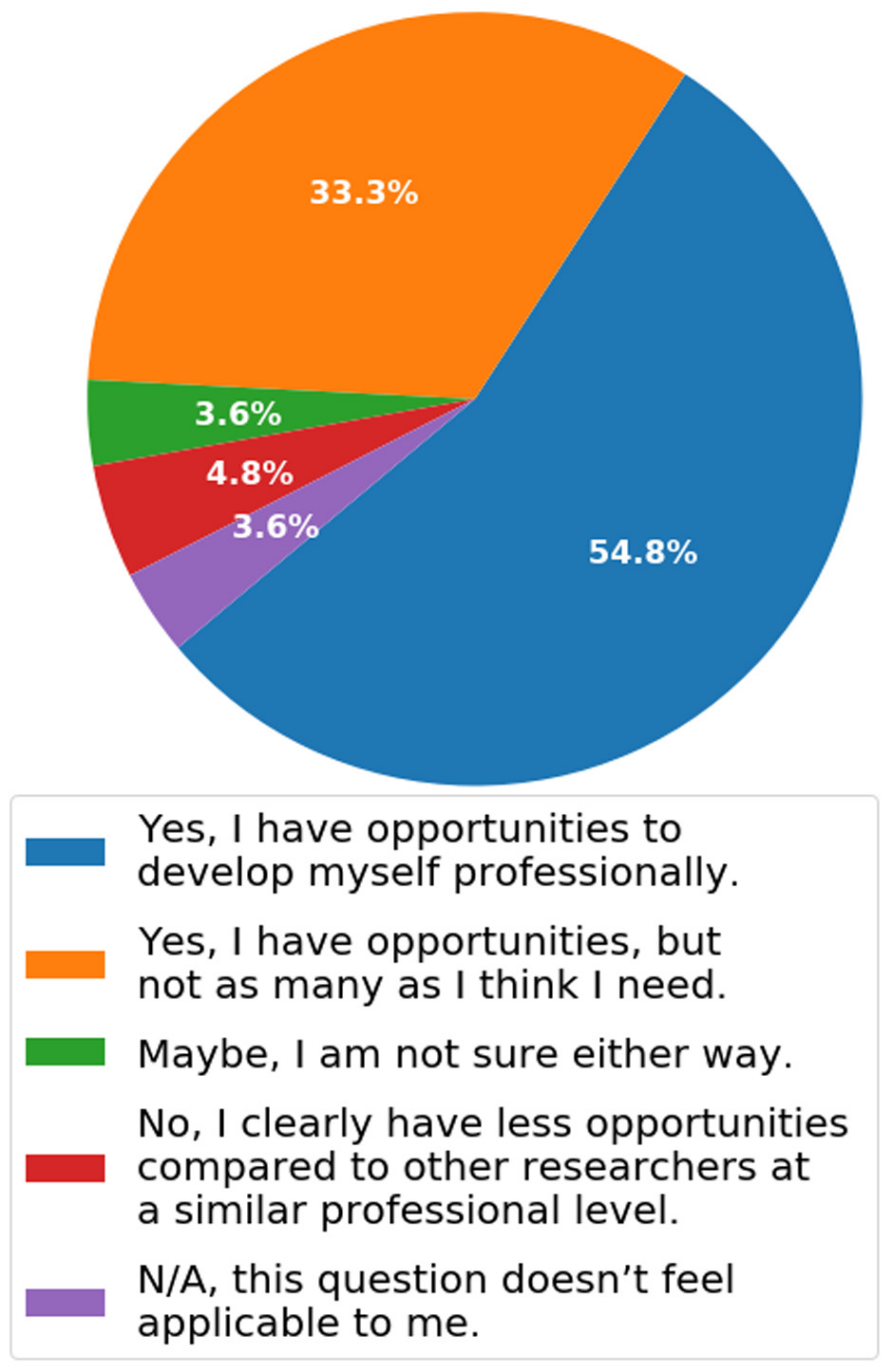
Bar chart representing answers to Q2.8. Data was pooled from across job descriptions for this question. Participants were able to select multiple answers.

***Q2.8: Have you found that your funding/university have included adequate resources for you to develop professionally (i.e. have been allowed and encouraged to apply for your own funding, attend conferences, network)?*** The answers from Q2.8 suggest that the majority (54.8%) of researchers in 2019 have sufficient money and opportunities to develop professionally ( Figure 12). In total, 33.3% believe more opportunities are needed, but that there are some opportunities available for them. This is a fairly good representation of how the existing funding opportunities support career development in the life sciences through conference attendance, networking and opportunities to apply for certain kinds of grants.

**Figure 13.**
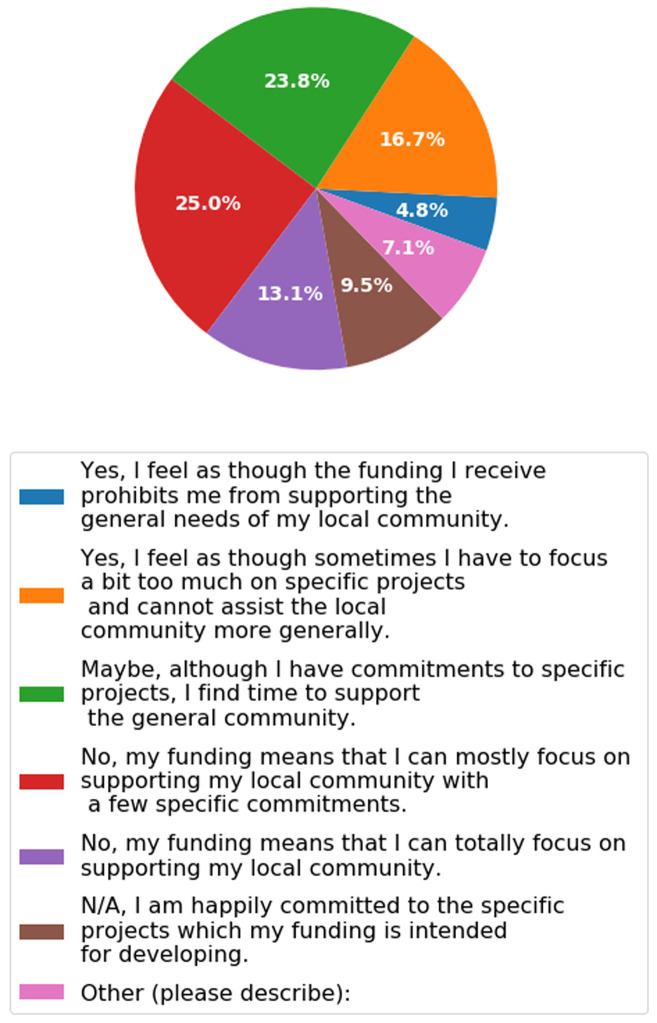
Bar chart representing answers to Q2.9. Participants were able to select only one answer. Data was pooled from across job descriptions for this question.

***Q2.9: Statement: Due to the sometimes mixed and ad-hoc nature of funding sources it is possible that one’s source of funding has been detrimental to your efforts to support your local community as a whole (i.e. you have not been able to assist people with general problems because of commitments to a particular funding source). Question. Do you agree with the above statement?*** We asked Q2.9 to ascertain whether participants in their current position felt as though they have enough flexibility to address the needs of their institute. There replies were broad to this question with a range of answers representing a unique set of circumstances ( Figure 13). In general, most participants felt that they could assist the local community to some level of capacity with only 4.8% saying that they couldn’t address the needs of the local community, due to tight funding restrictions. This shows that once funding has been obtained then there is sufficient flexibility within the funding’s remit to support the needs of the local community.

**Figure 14.**
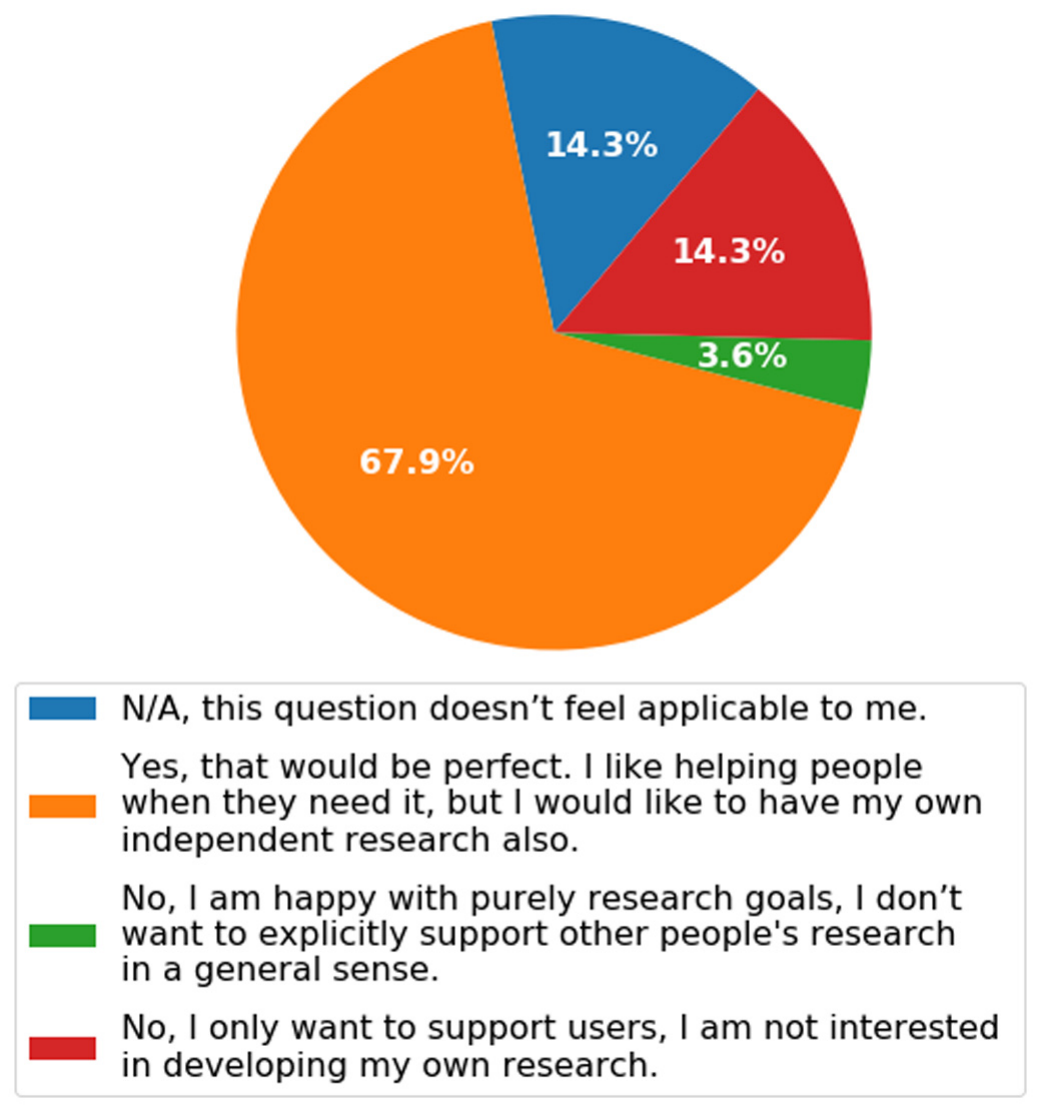
Pie chart representing the answers to Q2.10. Participants were able to select only one answer. Data was pooled from across job descriptions for this question.

***Q2.10: Statement: Individual researchers, in terms of their career, are feeling as though they must choose in a binary sense between pursuing independent research or a service/support role. Question: Would you be interested in funding which financially assisted you to support your local community (i.e. training and assisting in other people’s projects), but also allowed some time to pursue your own independent research interests (similar to a fellowship)?*** The answer to this question was very clear ( Figure 14); a job role whereby you can have your own ongoing research, but also support the community directly, is seen as ideal. Most Bioimage Analysts on an academic track realise that for good uptake of their approaches they need to work directly with users and support their ongoing work. Conversely, if novel techniques are developed these should be allowed to form the genesis of academic progression, even if the work was done in support of another researcher’s research project. This mixed model of research and support would be a good definition for establishing and representing Bioimage Analysts as a discrete entity, a system which allows them to have an identity in both the academic and technical settings. In addition to the set answers, we allowed the participants to also input their own answers. These are included in the *Extended data* ^3^ and echo the results from the main question.

## Conclusions

Currently the research environment in the UK does not formally recognise Bioimage Analysts as a discrete entity, in terms of its funding opportunities, and career development. Researchers who perform this role are a mixed-bag with a variety of backgrounds and job descriptions (Q2.4). The discipline is new and so this is understandable, however it is clear that as the discipline grows, to retain top staff it is important that this discipline is adequately recognised and funded. Although there is flexibility in the system that does allow individuals who are doing bioimage analysis to exist, and train (Q2.8), it doesn’t do enough to support their long-term career goals (Q1.5).

Neubias did a great job of defining what a Bioimage Analyst is, and with this definition it becomes easier for research bodies and the community to recognise and fund scientists within this role. We hope however that the Bioimage Analyst job description retains its fluidity and does not get defined purely as an academic or as a technical role. Due to the ad-hoc nature of the role up and to this point, it has allowed a certain degree of flexibility in how individuals have fulfilled this responsibility. Because someone decides to leave the academic track in favour of a role which is more aligned with support should not mean the outcomes of their work prohibit them from perhaps returning to an academic track at a later date. Quite the opposite, we want to encourage individual scientists to support the community more. The best way to encourage top-researchers to disseminate their specialist knowledge is through encouraging them with research money predicated on them also providing training and support content. We also recognise that scientists crossing from more wet-laboratory practises may want to spend time in a facility, to develop their technical skills, before going back into an academic career track. Individuals should be encouraged to do this and so metrics of productivity should include co-authorships, software maintenance and training. Therefore, we think it would a good idea to introduce fellowships which encourage a significant amount of support in the role. Furthermore, we think that facility staff should be allowed to apply for grants which mean they can offset their time from support to allow them to develop their academic independence, whether it be through maintaining software they have developed, or allowing them to pursue a research question based on an interest which has emerged from their work in the facility. The vast bulk of participants in this questionnaire agree with this sentiment of mixed research/support fellowships (Q2.10).

In summary this report has documented and summarised the results of two questionnaires designed to probe and understand the research community in the UK who are currently engaged in some form of bioimage analysis. It is essential that research bodies properly recognise this discipline and do so by providing long-term career possibilities which are suited to this role. By supporting initiatives involving bioimage analysis and also through recognising the efforts of groups like Neubias and the IAFIG, funders, universities and research bodies will yield the benefits from an emerging and highly productive community.

## Data availability

### Underlying data

Zenodo: Underlying data from two questionnaires used to understand the research climate of Bioimage Analysts in the UK between 2016–2019, http://doi.org/10.5281/zenodo.4605416 ^3^.

This project contains the following underlying data:

- Questionnaire analysis 1.ipynb (Jupyter Notebook, Python 3.5+)
- Questionnaire analysis 2.ipynb (Jupyter Notebook, Python 3.5+)
- Questionnaire 1+2.zip (zip file containing questionnaire 1+2 raw data (with email addresses removed))

### Extended data

Zenodo: Underlying data from two questionnaires used to understand the research climate of Bioimage Analysts in the UK between 2016–2019, http://doi.org/10.5281/zenodo.4605416 ^3^.

This project contains the following extended data:

- Extended Data 1: Questions asked for Questionnaire 1 and 2 using SurveyMonkey (Extended Data.pdf).
- Extended Data 2: Questionnaire 2 respondents localized by UK (and Ireland) university affiliation. Location determined from academic email address suffix (Extended Data.pdf).
- Extended Data 3: The ’other’ points raised as an optional comment field to Q2.10 (Extended Data.pdf).

Data are available under the terms of the Creative Commons Attribution 4.0 International license (CC-BY 4.0).
