# Supplementary material for: Summary of two questionnaires designed to understand the research climate for Bioimage Analysts in the UK between 2016-2019
Source: F1000Res. 2021 Apr 6;10:276. [Version 1] doi: 10.12688/f1000research.51794.1 (PMC8080981; doi:10.12688/f1000research.51794.1)
Supplement: Peer review history

# Peer review: Reviewer response for version 1

**DOI:** 10.5256/f1000research.54999.r82873

**Published:** 2021-04-22

**Authors:** Martin L. Jones

**Affiliations:** 1 Electron Microscopy Science Technology Platform, The Francis Crick Institute, London, UK

This article presents the results of two surveys carried out across the UK's community of researchers and facility staff using image analysis as part of their work. The analyses of the responses are presented along with the anonymised raw data. The "Extended Data" provides free text supplied by respondents, providing useful additional comments.

 The responses provide very useful insights into the current situation of the role of bioimage analysts in the UK and the analysis of the responses is thorough and well presented. Taken along with the results of the referenced NEUBIAS survey, this kind of information will hopefully raise the profile of bioimage analyst as a unique role, as well as seed discussions both in the bioimage analyst community and the broader research community, regarding the development and sustainability of careers in the area. It feels to me as though there are many parallels with the Research Software Engineering community, which may provide some useful roadmaps for the development of the bioimage analyst community. Community engagement and advocacy appear to have been key in the establishment of the RSE role and hopefully articles like this and the referenced NEUBIAS survey results will help bioimage analysts along a similar path! 

 Some specific minor comments:

- In Abstract: Methods: "Respondents were collected" should be "Responses were collected" or "Respondents' answers were collected".
- NEUBIAS should be capitalised throughout.
- COST should be capitalised throughout.
- In Methods, "Royal Software Engineers" should presumably be "Research Software Engineers"?
- In Methods, "In questionnaire 1 a Wordcloud (1)" should be "… a Wordcloud (Figure 1)" and ideally hyperlinked as elsewhere.
- Inconsistent capitalisation "questionnaire 1 vs Questionnaire 2" at a few places through the document.
- Given the hopefully international readership, it might be worth stating somewhere you're using dd/mm/yyyy date format, since many countries use different formats.
- In the Twitter response, does "6 Tweets" mean quote-tweets? 

 

 Results and discussion:

- Q1.1 - the closing single quotes are incorrectly formatted (and in a few other places).
- Q1.2 - opening single quotes are incorrectly formatted.
- Q1.3 - "either as a Bioimage Analyst, Specialist Image Analyst but a few used the term Software Engineers" should be "either as a Bioimage Analyst **or** Specialist Image Analyst, but a few used the term Software Engineers".
- Q1.5 - "…their obvious job description" doesn't sound right - obvious to whom? Should this be "stated job description"?
- Q2.4 makes the important distinction between facility- and research-centric responses and the fact that bioimage analyst is highly and equally represented in both groups supports the conclusions drawn.
- Q2.5 - I'm not sure that a good reference exists, but "This is fairly typical in the life sciences…" should ideally be backed up with a reference, or it should be stated if this is anecdotal.
- Q2.6 - it might be useful to list the funders in descending order of number of mentions (and state that it is so ordered). Also expanding the abbreviations would be useful for non-UK based readers as most are national funders only.
- Q2.9 - typo "There replies were broad". It would perhaps be interesting to see the breakdown of this question according to facility/research status as well, one might suspect that facility staff are more likely able to explicitly support their community.
- Q2.10 - it'd be interesting to see this split according to research/facility as well. As the main identified "track" subdivision, it may be useful to have separate results for all questions in fact (where applicable), either in the main document or in the Extended Data, with reference in the main text where differences are appreciable. Most readers will naturally fall into one camp or the other, so being able to quickly make comparisons may be beneficial, whereas questions with more polarised answers may give an artificially watered down consensus when combined. It feels to me as though these surveys have identified this research vs. facility difference as a key and interesting area and so full exposition of the results would likely be useful.

 

 Reference 1 should be updated with the new DOI: 10.5281/zenodo.4648077

Is the work clearly and accurately presented and does it cite the current literature?

Yes

If applicable, is the statistical analysis and its interpretation appropriate?

Yes

Are all the source data underlying the results available to ensure full reproducibility?

Yes

Is the study design appropriate and is the work technically sound?

Yes

Are the conclusions drawn adequately supported by the results?

Yes

Are sufficient details of methods and analysis provided to allow replication by others?

Yes

Reviewer Expertise:

Bioimage analysis, microscope development, software development, electron microscopy

I confirm that I have read this submission and believe that I have an appropriate level of expertise to confirm that it is of an acceptable scientific standard, however I have significant reservations, as outlined above.

## References

[1] : A Survey on Bioimage Analysis Needs, 2015. *Zenodo*.2015; 10.5281/zenodo.4648077 10.5281/zenodo.4648077 Reference source

# Peer review: Reviewer response for version 1

**DOI:** 10.5256/f1000research.54999.r82872

**Published:** 2021-04-19

**Authors:** Julia Fernandez-Rodriguez

**Affiliations:** 1 Centre for Cellular Imaging, University of Gothenburg, University of Gothenburg, Sweden

The paper has done an excellent job first to highlight the need for Bioimage Analysts, and second show clearly one of the important problems of the field: the funded scheme. Without a clear support from the funders (universities and research bodies) it will be impossible to provide career possibilities for these professionals. Currently the research environment in the UK, but also in the rest of Europe, does not formally recognise Bioimage Analysts as a discrete entity, in terms of its funding opportunities, and career development.

 It is also quite interesting that the respondents find a good model for funding the mixture of: their independent research interest and the financial means to support the local community with bioimage analysis. This also seems a good strategy to define a Bioimage Analysts. This founding model will allow them to have an identity in both the academic and technical settings.

 I do think the paper has to be indexed to show the problems about the recognition of the Bioimage Analysts and to provide long-term career possibilities.

Is the work clearly and accurately presented and does it cite the current literature?

Yes

If applicable, is the statistical analysis and its interpretation appropriate?

Yes

Are all the source data underlying the results available to ensure full reproducibility?

Yes

Is the study design appropriate and is the work technically sound?

Yes

Are the conclusions drawn adequately supported by the results?

Yes

Are sufficient details of methods and analysis provided to allow replication by others?

Yes

Reviewer Expertise:

Head of an Imaging Facility, background in Cell and Molecular Biology

I confirm that I have read this submission and believe that I have an appropriate level of expertise to confirm that it is of an acceptable scientific standard.

# Peer review: Reviewer response for version 1

**DOI:** 10.5256/f1000research.54999.r82875

**Published:** 2021-04-12

**Authors:** Julien Colombelli

**Affiliations:** 1 Advanced Digital Microscopy, Institute for Research in Biomedicine (IRB Barcelona), Barcelona, Spain

The author provides a report on two surveys aimed to capture a snapshot for the status of Biomage analysis as a field of research in the UK, including the research/service activities of its stakeholders, the staff distribution and funding perspectives, and their perception on the adequacy of funding in this field.

 Overall, the results are very interesting and provide, although at the national level for UK and with a relatively contained numbers of respondents, a rather precise snapshot that no other national or international survey, to date and with data, has reported to support a discussion on the place of bioimage analysis and bioimage analysts in Life Science. 

 In general, the paper has a great value for the community and should stimulate other countries to perform similar studies to capture invaluable data on the progress made in Life science towards supporting Biomage Analysis, and its staff, as a critical activity that needs better definition and recognition.

 I recommend minor clarifications detailed below.

**General comments on Methods:**

- Were all questions optional or mandatory?
  - See below for Q1.5 where it is relevant to know this.
- The sum of % sometimes exceeds 100%, hence the author should check the rounding for each value in the graphs.
- When respondents were given the chance to answer multiple options, the choice of a Pie chart in % is questionable. Probably (see specific answers) those answers categories that aim to be discussed should be pulled out as well on their individual frequency of answer, for which a % distribution would make more sense.

**General comments on: Results and Discussion:**

- Q1.1: treating all words in the respondent's answers separately may denature the meaning of the answers.
  - Also, duplicates in the word cloud could dilute specific answers (capital letter issue?).
  - If possible, it would be interesting to get another layer of interpretation to see for example how many general categories of respondents are represented, e.g. industry vs. public, management level vs. staff vs. PhD student & postdocs, etc...
- Q1.4 is not clearly analysed in my opinion.
  - The question aims at distributing the work time of respondents spent on activities identified in Q1.3, but the weighting factor could bias the total sum of time.
  - The options given to respondents to quantify their time were: all of their time is valued 100%(8), most: 75%(6), half: 50%(4), some: 12.5%(1), none: 0%(0)
  - However, if a person answers (see e.g. n30 in Zenodo data):
  - Image Analysis User: Some.
  - Bioimage Analyst: Some.
  - Then the total estimated time which is counted here is 25%.
  - Problem: we don't know what that person does with the rest (75%) of the worktime, and the analysis of the data does not seem to count it. Hence the global impression (pie chart, figure) is misleading, suggesting for example that all respondents spend, on average, only 34.2% of their time on other tasks than Image analysis, which is wrong. Respondent n30 spends 75% of time on something else, but it doesn't seem to be counted.
  - Each answer therefore sums a different amount of time, which makes the final % distribution of the figure somehow biased.
  - I would like to see, therefore, how much absolute time of the respondents is spent on Image analysis, so as to support the conclusion of the paragraph. This could be done by estimating a new category "rest of the time", possibly to be fused with "Other" when respondents have quantified this category for their working time.
- Another doubt, with the same origin of bias I believe:
  - In the answers, how can the 35.4% of subjects answering Q1.3 with "Image Analysis Users" convert into 2.3% of time spent on "Using Image Analysis"?
  - Assume those 35.4% would spend minimally "some time", then the % should be **at least **12.5% of 35.4% = 4.4%.​​​​​
  - Here, possibly, a crossed plot between Q1.3 and Q1.4 would be very informative to see how users spend their time on analysis, how many are intensive users (half of their time and above), etc... for each category.
- Q1.5: There are two questions in one.
  - The author should clarify how the distinction of "who is interested in pursuing image analysis as potential career" is made: is it by answering the question?
  - Was the question optional?
    ​​​​​​​
- Q2.4:
  - This question reports very interesting results, redistributed against facility or research-oriented groups categories.
  - However, given that the respondents could select more than one option, the distribution of the results in % is questionable. Here, to support the conclusions, the author could draw the absolute % against the total # of respondents for each specific answer: e.g. how many of the respondents have ticked "Trainer" respect to the total number of respondents in the specific category. I expect those individual % to increase for each response, hence reinforcing the conclusions.
    ​​​​​​​
- Q2.6: Probably, the acronyms should be expanded in the legend so as to ease the reading for non-specialist readers (e.g. probably out of UK).
  - Also, readers not aware of the different funders in the UK will probably have a hard time understanding the different nature of the funds (whether for research projects, infrastructure or other). Colors in the graph could orient the reader and provide a more detailed qualitative interpretation here.
  - Although more difficult to draw, but maybe accessible in the current data, it could be interesting to get a sense for whether public vs. private funding contribute more or less to Bioimage Analysis.
- Q2.7: This question is very important and unique, it captures the stability of jobs in Bioimage Analysis. The answers to this question could, ideally, be discussed in the discussion part of the paper, to try to evaluate or compare to other communities/professional groups in Life Science and see if Bioimage Analysis related positions suffers a bias towards short term contracts.
- Q2.8: Here again, a % distribution for a multiple choice question yields a Pie chart that may not be accurate.
  ​​​​​​​
- Q2.10: It could be interesting to redistribute the answers on the facility vs. research groups category.
  - The extended data is not easy to "read/go through", here some extracts of the "own answers" could be interesting to read, if they provide interesting arguments/examples.
  - In general, if feasible, it would be interesting to do this exercise on all questions, e.g. setting "inset" pie charts, in each figure where the answers where "data was pooled across job descriptions". Even if the data would not be discussed, it would release the interested reader (from one category or the other) from the duty to get into python scripting against the raw data.

**Conclusions:**

- Are there examples of grants/funds/fellowships dedicated to mixed research/support, in UK or beyond? It is a major topic for this paper and some context/background/example would probably help illustrating the current possibilities, or reinforcing the current lack of such funding (if there is no example to feature).

**References: **are very few. Are there any equivalent study/survey reports in the field of e.g. imaging facilities or other types of facilities that could be cited and where, maybe, data is similar and can serve to elaborate more discussion ?

**Specific comments:**

- Please refer to NEUBIAS with capital letters.

**Refs:**

1. This reference has a version on Zenodo: Miura (2021 ^1^).
2. Better to use www.neubias.org as a landing pag for NEUBIAS.

Is the work clearly and accurately presented and does it cite the current literature?

Partly

If applicable, is the statistical analysis and its interpretation appropriate?

Partly

Are all the source data underlying the results available to ensure full reproducibility?

Yes

Is the study design appropriate and is the work technically sound?

Yes

Are the conclusions drawn adequately supported by the results?

Yes

Are sufficient details of methods and analysis provided to allow replication by others?

Yes

Reviewer Expertise:

Light Microscopy

I confirm that I have read this submission and believe that I have an appropriate level of expertise to confirm that it is of an acceptable scientific standard, however I have significant reservations, as outlined above.

## References

[1] : A Survey on Bioimage Analysis Needs, 2015. *Zenodo*.2021; 10.5281/zenodo.4648077 10.5281/zenodo.4648077 Reference source
